# Pre-treatment of human umbilical cord-derived mesenchymal stem cells with interleukin-6 abolishes their growth-promoting effect on gastric cancer cells

**DOI:** 10.3892/ijmm.2014.2019

**Published:** 2014-12-02

**Authors:** MEI WANG, JIE CAI, FENG HUANG, MENGCHU ZHU, QIANG ZHANG, TINGTING YANG, XU ZHANG, HUI QIAN, WENRONG XU

**Affiliations:** Key Laboratory of Medical Science and Laboratory Medicine of Jiangsu Province, The Affiliated Hospital, School of Medicine, Jiangsu University, Zhenjiang, Jiangsu 212013, P.R. China

**Keywords:** interleukin-6, human umbilical cord-derived mesenchymal stem cells, gastric cancer

## Abstract

The inflammatory microenvironment contributes to cancer development and progression. Mesenchymal stem cells (MSCs), as important stromal cells, may be ‘educated’ by the inflammatory microenvironment to support the development of gastric cancer. Cytokines are a key component of cancer-related inflammation. Interleukin (IL)-6, as an inflammatory cytokine, has multiple roles in cancer. However, whether MSCs can be ‘educated’ by IL-6 to support gastric cancer remains unknown. In the present study, we focused on the phenotype and function of human umbilical cord-derived MSCs hUC-MSCs pre-treated with IL-6 in gastric cancer. We found that the protein levels of α-smooth muscle actin (α-SMA) were upregulated, and phosphorylated nuclear factor (NF)-κB protein levels were downregulated in the hUC-MSCs pretreated with IL-6, as shown by western blot analysis. The levels of tumor-promoting cytokines, including chemokine (C-C motif) ligand 5 (CCL5), platelet-derived growth factor-BB (PDGF-BB), monocyte chemoattractant protein-1 (MCP-1) and tumor necrosis factor α(TNFα), were markedly reduced in the hUC-MSCs following treatment with IL-6, as shown by RT-qPCR. In *in vitro* experiments*,* we co-cultured MSCs with N-methyl-N’-nitro-N-nitrosoguanidine (MNNG)-transformed GES-1 gastric epithelial cells or SGC-7901 gastric cancer cells. Transwell and colony-forming cell assays revealed that the hUC-MSCs significantly promoted gastric cellular migration and proliferation. However, following treatment with IL-6, the hUC-MSCs had no growth-promoting effect on the gastric epithelial cells and gastric cancer cells. In *in vivo* experiments, we co-transplanted MSCs and SGC-7901 cells into nude mice in order to establish a nude mouse model of gastric cancer. The hUC-MSCs significantly promoted the growth gastric tumors through the promotion of cell proliferation and the inhibition of cell apoptosis. On the contrary, pre-treatment with IL-6 provided the hUC-MSCs with the ability to inhibit cell proliferation and significantly induce cell apoptosis. Taken together, our findings indicate that pre-treatment with IL-6 significantly abolishes the ability of hUC-MSCs to promote gastric epithelial cell proliferation and migration and provide new insight into the effects of the inflammatory cytokine, IL-6, on the tumor-promoting ability of MSCs and its role in gastric cancer.

## Introduction

Solid tumors contain many different cellular components in addition to tumor cells, including fibroblasts, lymphocytes, dendritic cells, macrophages and other myeloid cells. Complex interactions between the stromal cells in this microenvironment regulate tumor development and progression (1). The stromal cells and mediators of inflammation form a major part of the epithelial tumor microenvironment (2,3). Gastric cancer is a classic model of chronic inflammation preceding malignancy, and the tumor-promoting inflammatory microenvironment promotes the malignant transformation process (4).

The cancer-related inflammatory microenvironment covers several types of stromal cells, which include macrophages, carcinoma-associated fibroblasts (CAFs), leukocytes and mesenchymal stem cells (MSCs). Among these stromal cell types, MSCs have been strongly associated with the progression of cancer (5,6). We have previously isolated MSC-like cells from gastric cancer tissue (GC-MSCs) and adjacent normal gastric tissue (GCN-MSCs) (7,8). We have also previously found that the ability of GC-MSCs to promote gastric cancer was stronger than that of GCN-MSCs (9). GC-MSCs secreted higher levels of inflammatory cytokines than the GCN-MSCs. This finding suggests that GC-MSCs and GCN-MSCs are representative of different stages of cancer-related inflammatory conditions. The inflammatory microenvironment plays an important role in the conversion of MSCs into tumor-supporting cells.

Accumulating evidence indicates that MSCs co-cultured with cancer cells or treated with cancer-cell culture-conditioned medium can be activated to assume the tumor-promoting phenotype (10). Recently, Ren *et al* (11) reported that tumor stromal cells can endow normal stromal cells with tumor-promoting properties. In a previous study of ours, we treated human umbilical cord-derived MSCs (hUC-MSCs with gastric cancer cell-derived exosomes and found that the hUC-MSCs differentiated into CAFs (12). In order to mimic gastritis infection microenvironment better, we infected hucMSC (hUC-MSCs with *Helicobacter pylori* (*H. pylori*) and found that the hUC-MSCs also differentiated into CAFs and promoted epithelial-mesenchymal transition in gastric epithelial cells (13). We have also previously found that the hUC-MSCs activated by inflammatory macrophages contribute to human gastric carcinogenesis through nuclear factor (NF)-κB activation (14). These findings suggest that hUC-MSCs can be activated to acquire the cancer-promoting phenotype.

Gastric cancer cell-derived exosomes, *H. pylori* and macrophages are important constituents of cancer-related inflammation. Notably, inflammatory cytokines are mediators that regulate a broad range of processes involved in the pathogenesis of cancer (15). Among these cytokines, interleukin (IL)-6 has been proven to be a key growth-promoting and anti-apoptotic inflammatory cytokine and is also one of the effector signals in the promotion of carcinogenesis (16–18). Furthermore, IL-6 acts as an essential factor mediating the interaction between MSCs and cancer cells (18–20). Recently, Sung *et al* (21) revealed that the upregulation of IL-6 in bone marrow-derived MSCs triggered a reactive stromal response to prostate cancer. Whether IL-6 in an inflammatory microenvironment acts on MSCs and induces them to acquire the cancer-promoting phenotype remains unknown.

In the present study, we pre-treated hUC-MSCs with IL-6 and investigated the phenotype and function in gastric cancer development *in vitro* and *in vivo*. The present study provides new evidence on whether the inflammatory cytokine, IL-6, can ‘educate’ hUC-MSCs to support the development of gastric cancer.

## Materials and methods

### hUC-MSC isolation and culture

hUC-MSCs were obtained and the characteristics of the isolated hUC-MSCs were investigated as previously described (22). Briefly, the hUC-MSCs were photographed for the analysis of their morphological appearance. Surface antigens, including FITC-CD34, CD71, HLA-DR, PE-CD29, CD38, CD44, CD105 and HLA-I, were detected and analyzed by flow cytometry. Von Kossa staining and Oil Red O staining were used to evaluate their osteogenic differentiation and adipogenic differentiation potential, respectively. hUC-MSCs at passage 3 were used for the experiments. All experimental protocols were approved by the Ethics Committee of Jiangsu University, Zhenjiang, China.

### Cell lines and culture

The GES-1 gastric epithelial cell line and the SGC-7901 gastric cancer cell line were maintained and cultured in Dulbecco’s modified Eagle’s medium (DMEM; Life Technologies, Grand Island, NY, USA) containing 4,500 mg/l glucose (HG-DMEM), L-glutamine and 110 mg/l sodium pyruvate supplemented with 10% fetal bovine serum (FBS; Life Technologies). The cells were incubated at 37°C in humidified air with 5% CO_2_. The GES-1 cells were treated with 2×10^−5^ mol/l of N-methyl-N’-nitro-N-nitrosoguanidine (MNNG) (Sigma-Aldrich Co., LLC., St. Louis, MO, USA) for 24 h and most of the cells died in the following days. One week later, colonies of the transformed cells were formed and were used as an *in vitro* model of gastric precancerous lesions.

### Pre-treatment of hUC-MSCs with IL-6

One day before treatment, the hUC-MSCs were trypsinized and counted. The hUC-MSCs (4×10^4^) were plated in a 6-well plate (Corning Inc., Corning, NY, USA) and allowed to adhere overnight. The culture medium of the hUC-MSCs was discarded and replaced with fresh culture medium containing 50 ng/ml of human recombinant IL-6 (R&D Systems Inc., Minneapolis, MN, USA). After 48 h, the hUC-MSCs were used for the following experiments.

### RNA isolation and reverse transcription-quantitative PCR (RT-qPCR)

Total RNA was extracted from the cells using TRIzol^®^ reagent (Life Technologies) according to the manufacturer’s instructions, and an equal amount of RNA was reverse transcribed using the RevertAid First Strand cDNA Synthesis kit (Fermentas, Glen Burnie, MD, USA). RT-qPCR was performed to detect the changes in mRNA expression using the SYBR-Green I Real-Time PCR kit (Vazyme Biotech Co., Ltd., Nanjing, China) and the Bio-Rad fluorescence thermal cycler (Bio-Rad Laboratories, Hercules, CA, USA). The relative mRNA expression was normalized to the insert control gene, β-actin, according to the manufacturer’s instructions. The primers used in the present study were produced by Invitrogen (Life Technologies). All primer sequences and RT-qPCR conditions are listed in Table I.

### Luminex assay

The human Cytokine and Chemokine Magnetic Bead Panel kit (#HCYTOMAG-60K) (Merck Millipore, Darmstadt, Germany) was designed to detect granulocyte colony stimulating factor (G-CSF), IL-10, platelet-derived growth factor-BB (PDGF-BB), IL-6, IL-8, monocyte chemoattractant protein-1 (MCP-1), tumor necrosis factor α (TNFα) and vascular endothelial growth factor (VEGF) in the supernatant from hUC-MSCs and IL-6-pre-treated hUC-MSCs. All procedures were processed according to the manufacturer’s instructions. The signal was detected and analyzed using the Luminex 200 System (Merck Millipore).

### Western blot analysis

The primary antibodies used for western blot analysis were as follows: rabbit antibodies against phosphorylated (p-)signal transducer and activator of transcription 3 (STAT3; Cat. no. 11045), STAT3 (Cat. no.23220), p-NF-κB (Cat. no. 11014), NF-κB (Cat. no. 21012; all obtained from Signalway Antibody Co., Ltd., Baltimore, MD, USA), and α-smooth muscle actin (α-SMA; Cat. no. BS7000; Bioworld Technology, Louis Park, MN, USA). Following incubation with the secondary antibodies (Cat. no. 13012-2; Signalway Antibody Co., Ltd.), the signal was visualized using HRP substrate (Millipore, Billerica, MA, USA) and analyzed using MD Image Quant Software. GAPDH was used as the loading control.

### Co-culture model

The MNNG-transformed GES-1 cells or SGC-7901 gastric cancer cells were plated into the lower chamber of a 6-well plate for 8 h. The hUC-MSCs and the IL-6-pre-treated hUC-MSCs were then placed into the top chamber of Transwell plates (0.4-*μ*m pore size; Corning Inc.). The MSCs were seeded at a density of 1×10^5^ cells/well in 1.6 ml of complete DMEM medium. The GES-1 and SGC-7901 cells were seeded at a density of 1×10^5^ cells/well in 2.5 ml of HG-DMEM medium. The GES-1 and SGC-7901 cells were collected for analysis following indirect co-culture with the MSCs for 48 h. Cells cultured in medium only were used as the controls.

### Cell colony formation

Following co-culture for 48 h, the GES-1 and SGC-7901 cells were trypsinized and resuspended to a concentration of 1,000 cells/2 ml HG-DMEM with 10% FBS and were then incubated for 10 days. Colonies were fixed with methanol, stained with crystal violet and counted.

### Transwell migration assay

Following co-culture for 48 h, the GES-1 and SGC-7901 cells (1×10^5^ cells/well) were plated into the top chamber, and medium containing 10% FBS was placed into the bottom chamber of Transwell plates (8-*μ*m pore size; Corning Inc.). Following incubation at 37°C in 5% CO_2_ for 10 h, the cells remaining on the upper surface of the membrane were removed with a cotton swab. The top chamber cells were incubated for 10 h, and cells that did not migrate through the pores were removed using a cotton swab. Cells on the lower surface of the membrane were fixed with methanol and stained with crystal violet. The migration ability of the cells was determined by counting the cells under a microscope (SN:9G15626; Olympus, Tokyo, Japan) in at least 6 fields for each assay.

### Immunofluorescence staining

Following co-cultrure for 48 h, the SGC-7901 cells were washed 3 times with cold PBS, fixed with 4% paraformaldehyde for 20 min, permeabilized with 0.1% Triton X-100 for 5 min, blocked with 5% BSA and incubated with proliferating cell nuclear antigen (PCNA) primary antibody (Cat. no. BS6438; Bioworld Technology) at 4°C over night and followed by Cy3-conjugated anti-rabbit secondary antibody (Cat. no. C2306; Sigma-Aldrich). The cells were then stained with DAPI for nuclear staining, and images were acquired using a Nikon Eclipse Ti-S microscope.

### Animal model

BALB/c nude mice (4–5 weeks old) were purchased from the SLAC Laboratory Animal Center (Shanghai, China) and were randomly divided into 3 groups. The animals were maintained in accordance with institutional policies, and all experiments were performed with approval of the University Committee on the Use and Care of Animals of Jiangsu University. The animals were injected subcutaneously with untreated SGC-7901 cells alone, SGC-7901 cells together with hUC-MSCs or IL-6-pre-treated hUC-MSCs into the backside of mice. Tumors were surgically removed, photographed and weighed 4 weeks after injection.

### Immunohistochemistry

Formalin-fixed paraffin-embedded mouse tumor tissue sections were first deparaffinized in xylene and rehydrated through graded ethanol. Subsequently, the sections were boiled for 10 min in citrate buffer (pH 6.0, 10 mM) for antigen retrieval. Endogenous peroxidase activity was then inhibited by exposure to 3% hydrogen peroxide for 10 min. The sections were then blocked with 5% BSA (Boster Bioengineering, Co., Ltd., Wuhan, China) and incubated with properly diluted PCNA primary antibody (Bioworld Technology) at 37°C for 1 h. After the sections were washed with PBS, they were then incubated with diluted secondary antibody for 20 min. Finally, the sections were visualized with 3,3′-diaminobenzidine (DAB) and then counterstained with hematoxylin for examination under a light microscope (×100, SN:9G15626; Olympus). The terminal deoxynucleotidyl transferase-mediated dUTP-biotin nick end labeling (TUNEL) assay was conducted to measure cell apoptosis according to the manufacturer’s instructions (Boster Bioengineering, Co., Ltd.).

### Statistical analysis

All experiments were conducted at least in triplicate. Data were presented as the means ± standard error. Statistical analysis was performed using SPSS 11.0 software. Potential differences between groups with different treatments were determined using one-way ANOVA or an independent-sample t-test. A value of P<0.05 was considered to indicate a statistically significant difference.

## Results

### Phenotype of hUC-MSCs pre-treated with IL-6

**Figure 1:**
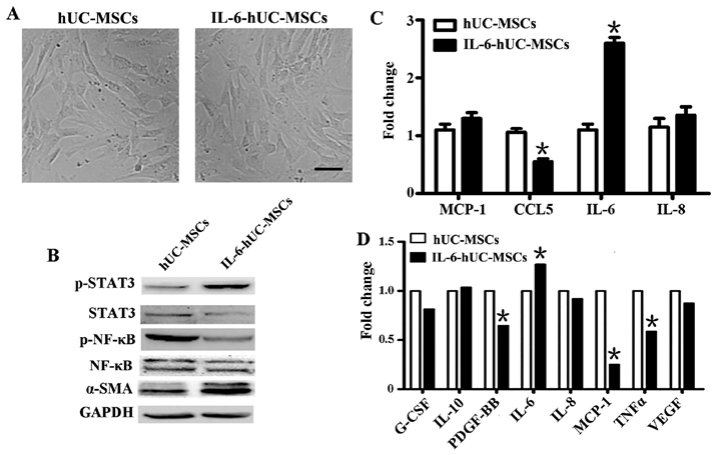
Phenotype human umbilical cord-derived mesenchymal stem cells (hUC-MSCs) treated with interleukin (IL)0-6 and inflammatory cytokines expression. (A) The morphology of hUC-MSCs and IL-6-hUC-MSCs; magnification, ×100; scale bar, 50 *μ*m. (B) Western blot analysis of protein expression of phosphorylated (p-)signal transducer and activator of transcription 3 (STAT3), STAT3, p-nuclear factor (NF)-κB, NF-κB and α-smooth muscle actin (α-SMA) in hUC-MSCs and IL-6-hUC-MSCs. (C) RT-qPCR of monocyte chemoattractant protein-1 (MCP-1), chemokine (C-C motif) ligand 5 (CCL5), IL-6 and IL-8 mRNA levels in the 2 aforememtioned types of cells. (D) Luminex assy of granulocyte colony stimulating factor (G-CSF), IL-10, platelet-derived growth factor-BB (PDGF-BB), IL-6, IL-8, MCP-1, tumor necsoris factor α (TNFα) and vascular endothelial growth factor (VEGF) levels in the supernatant from hUC-MSCs and IL-6-hUC-MSCs. IL-6-hUC-MSCs, hUC-MSCs pre-treated with IL-6 for 48 h. The values are expressed as the means and standard errors or the mean from at least 3 independent experiments. ^*^P<0.05.

The hUC-MSCs cultured at the third passage were treated with IL-6 for 48 h. The morphology of the hUC-MSCs pre-treated with IL-6 (IL-6-hUC-MSCs) did not differ from the spindle shape of the parental hUC-MSCs (Fig. 1A). In order to determine whether the hUC-MSCs can be activated by IL-6, the protein levels of phosphorylated (p-)STAT3, STAT3, p-NF-κB, NF-κB and α-SMA were determined by western blot analysis. The results revaled that STAT3 protein was significantly activated and phosphorylated by IL-6, accompanied by the increased levels of α-SMA protein (Fig. 1B). However, the phosphorylation levels of NF-κB, which is an important inflammatory transcription factor, were markedly decreased (Fig. 1B).

### Inflammatory cytokines secreted by hucMSCs pre-treated with IL-6

To evaluate whether NF-κB inactivation affects inflammatory cytokine secretion by IL-6-hUC-MSCs, we initially performed RT-qPCR to determine the mRNA levels of MCP-1, chemokine (C-C motif) ligand 5 (CCL5), IL-6 and IL-8 in the hUC-MSCs pre-treated with IL-6. We found that the mRNA levels of IL-6 were significantly increased, whereas the mRNA levels of CCL5 were significantly decreased. The other mRNA levels of the other 2 cytokines (MCP-1 and IL-8) did not exhibit any marked differences (Fig. 1C). To further analyze the cytokine profiles in the hUC-MSCs pre-treated with IL-6, the luminex analysis system was used to determine the content of several inflammation- and cancer-related cytokines in the cell culture supernatant, which included G-SCF, IL-10, PDGF-BB, IL-6, IL-8, MCP-1, TNFα and VEGF. We observed that the levels of PDGF-BB, MCP-1 and TNFα were markedly down-regulated in the supernatant of IL-6-hUC-MSCs. The level of IL-6 in the supernatant was upregulated, which was similar to the IL-6 mRNA level. The levels of the other cytokines (G-CSF, IL-10, IL-8, and VEGF) did not differ significantly between the hUC-MSCs and IL-6-hUC-MSCs (Fig. 1D).

### Pre-treatment of hUC-MSCs with IL-6 abolishes their growth-promoting effect on MNNG-transformed gastric epithelial cell in vitro

**Figure 2:**
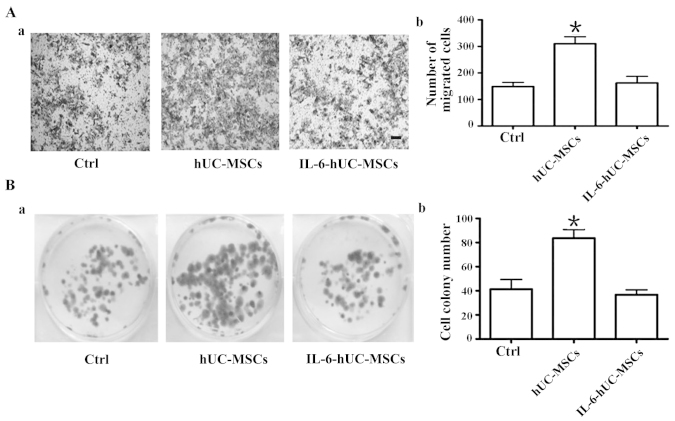
Effect of IL-6-human umbilical cord-derived mesenchymal stem cells (hUC-MSCs) on N-methyl-N’-nitro-N-nitrosoguanidine (MNNG)-transformed GES-1 gastric epithelial cells. (A) Transwell assay of MNNG-transformed GES-1 cell migration ability following co-culture with hUC-MSCs or IL-6-hUC-MSCs for 48 h. GES-1 cells alone served as control cells (Ctrl). (a) Representative photomicrograph of migrated cells. Magnification, ×100; scale bar, 50 *μ*m; (b) the number of migrated cells. (B) Plate colony formation assay for growth ability of GES-1 cells co-cultured with hUC-MSCs or IL-6 hUC-MSCs. (a) Representative photomicrograph of cell colony formation; (b) the number of cell colonies. IL-6-hUC-MSCs, hUC-MSCs pre-treated with IL-6 for 48 h. Bars indicate the means and standard error from at least 3 independent experiments. ^*^P<0.05.

MSCs have been strongly associated with cancer development and progression (5,6). Our results demonstrated that pre-treatment with IL-6 significantly inhibited inflammatory cytokines secretion by hUC-MSCs. In order to elucidate the role of IL-6-hUC-MSCs in gastric cancinogenesis, we treated GES-1 gastric epithelial cells with the carcinogenic agent, MNNG, and established MNNG-transformed GES-1 gastric epithelial cells (MNNG-GES-1). MNNG-GES-1 cells are representative of precancerous epithelial cells. We co-cultured the MNNG-GES-1 cells with the hUC-MSCs or IL-6-hUC-MSCs in Transwell plates for 48 h. Subsequently, we analyzed the migration and proliferation ability of the MNNG-GES-1 cells. Transwell assay revealed that the number of migrated cells in the IL-6-hUC-MSC group was smaller than that of the hUC-MSC group (Fig. 2A). Cell colony formation assay revealed that the size and number of the cell colonies in the IL-6-hUC-MSC group was smaller than that in the hUC-MSC group (Fig. 2B). There was no difference observed in the migration and proliferation ability of the MNNG-GES-1 cells between the control group and the IL-6-hUC-MSC group. The data indicated that the hUC-MSCs significantly promoted MNNG-GES-1 cell migration and proliferation. However, pretreatment with IL-6 abolished the growth-promoting effect of hUC-MSCs on MNNG-GES-1 cells.

### Pre-treatment with IL-6 strips hUC-MSCs of their growth promoting effect on gastric cancer cells through the inhibition of cell proliferation in vitro

**Figure 3:**
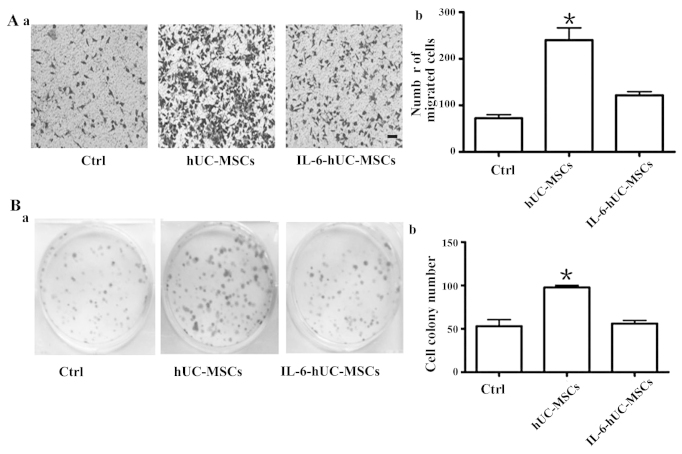
Effect of IL-6-human umbilical cord-derived mesenchymal stem cells (hUC-MSCs) on SGC-7901 gastric cancer cells. (A) Migration ability of SGC-7901 cells following co-culture with hUC-MSCs and IL-6-hUC-MSCs for 48 h. SGC-7901 cells alone served as controls (Ctrl). (a) Representative photomicrograph of migrated cells. Magnification, ×100; scale bar, 50 *μ*m; (b) the number of migrated cells. (B) Colony formation assay for growth ability of SGC-7901 cells co-cultured with hUC-MSCs or IL-6-hUC-MSCs. (a) Representative photomicrograph of cell colonies; (b) the number of cell colonies. IL-6-hUC-MSCs, hUC-MSCs pre-treated with IL-6 for 48 h. Bars indicate the means and standard errors from at least 3 independent experiments. ^*^P<0.05.

In order to determine the effect of IL-6-hucMSC on gastric cancer cells, we co-cultured the SGC-7901 gastric cancer cells with hUC-MSCs or IL-6-hUC-MSCs in a Transwell plate for 48 h. As shown in Fig. 3, the number of migrated cells in the IL-6-hUC-MSC group was smaller than that observed in the hUC-MSC group (Fig. 3A). The size and number of cell colonies in the IL-6-hUC-MSC group were smaller than those in the hUC-MSC group (Fig. 3B). The migration and proliferation ability of the SGC-7901 cells in the IL-6-hUC-MSC group was similar to that in the control group.

**Figure 4:**
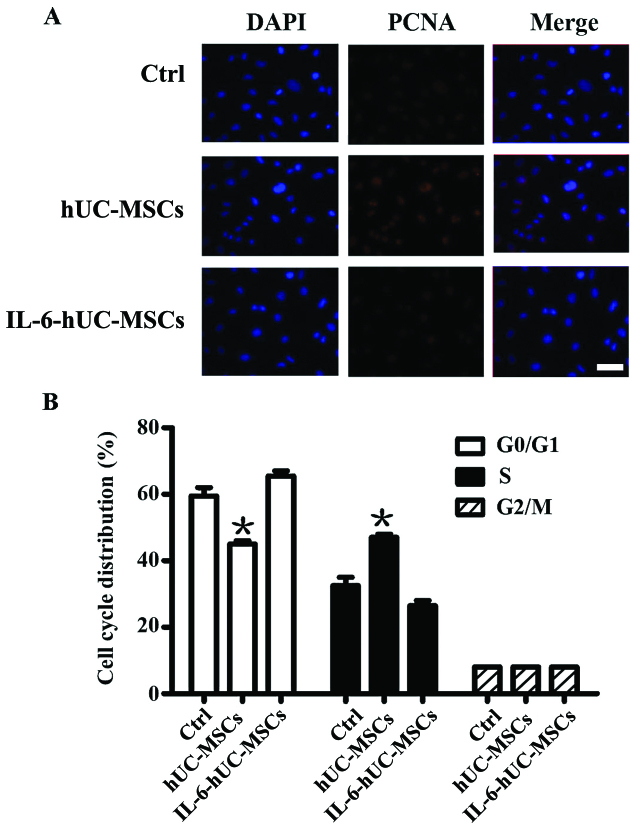
Proliferation and apoptosis-related proteins in SGC-7901 cells co-cultured with IL-6-human umbilical cord-derived mesenchymal stem cells (hUC-MSCs). (A) Immunofluorescence staining of the expression level of PCNA in SGC-7901 cells following culture with hUC-MSCs or IL-6-hUC-MSCs for 48 h. Magnification, ×200; scale bar, 50 *μ*m. (B) Cell cycle distribution of SGC-7901 cells following culture with hUC-MSCs or IL-6-hUC-MSCs. IL-6-hUC-MSCs, hUC-MSCs pre-treated with IL-6 for 48 h. Bars indicate the means and standard errors from at least 3 independent experiments. ^*^P<0.05.

To elucidate the effects of IL-6-hUC-MSCson the proliferation ability and cell cycle distribution of SGC-7901 cells, we determined PCNA expression and cell cycle progression by immunofluorescence staining and flow cytometry, respectively. The results revealed that PCNA expression was upregulated in the hUC-MSC group, wherease its expression level in the IL-6-hUC-MSC group was similar to that in the control group (Fig. 4A). Cell cycle analysis revealed that, compared to the control group, the percentage of SGC-7901 cells in the S phase was evidently increased in the hUC-MSC group and slightly decreased in the IL-6-hUC-MSC group (Fig. 4B). These data suggest that the hUC-MSCs significantly promote gastric cancer cell migration and proliferation. Pre-treatment with IL-6 stripped the hUC-MSCs of their growth-promoting effect on gastric cancer cells.

### Pre-treatment of hUC-MSCs with IL-6 abolishes their growth-promoting effect on SGC-7901 gastric cancer cell-derived tumor xenografts in vivo

**Figure 5:**
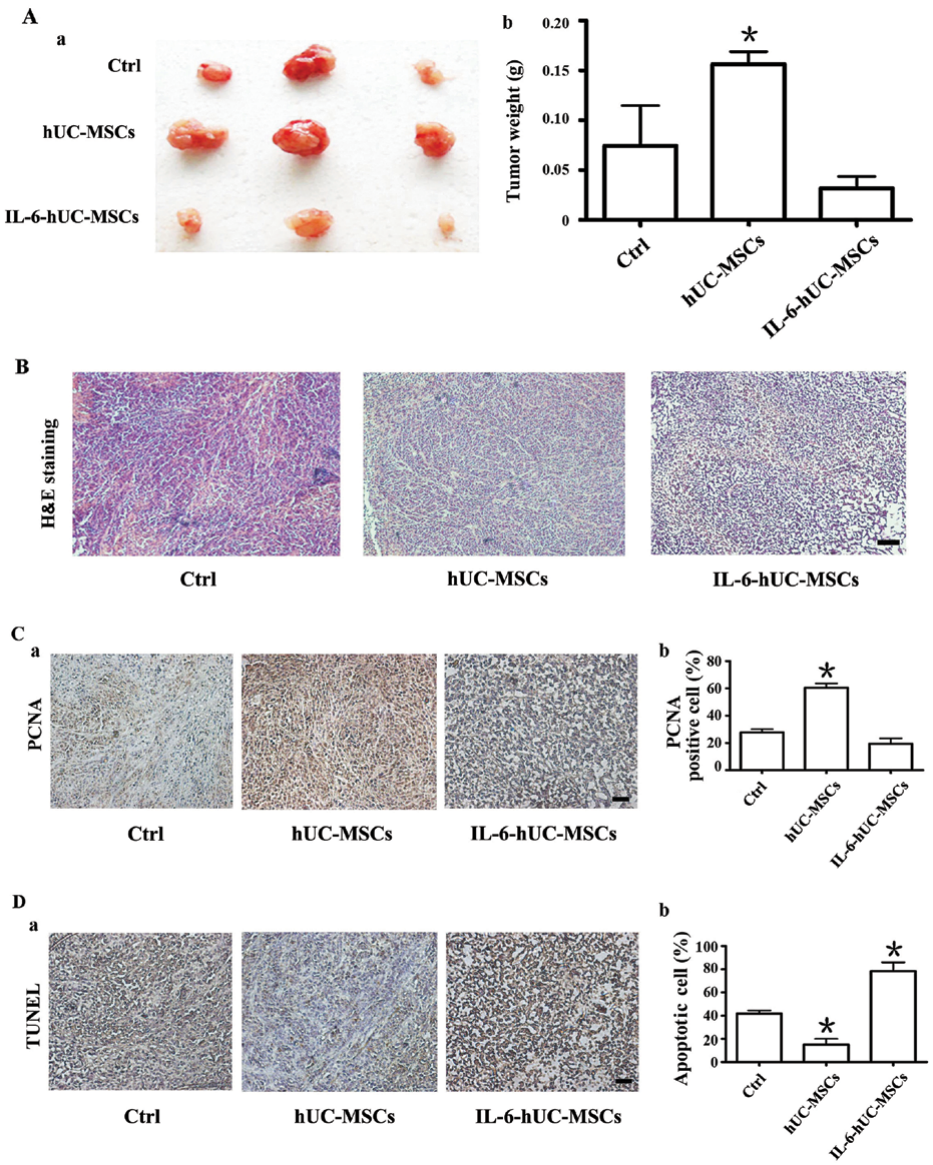
Role of IL-6-human umbilical cord-derived mesenchymal stem cells (hUC-MSCs) in SGC-7901 gastric cancer cell-derived tumor xenograft growth *in vivo*. (A-a) Representative photograph of tumor tissues; (b) tumor tissue weight. (B) H&E staining of tumor tissue. (C) Immunohistochemical analysis of PCNA expression in tumor tissues. (a) Representative photomicrograph; (b) percentage of proliferating cell nuclear antigen (PCNA)-positive cells. (D) TUNEL assay of apoptosis cells in tumor tissue. (a) Representative photomicrograph; (b) percentage of apoptotic cells. IL-6-hUC-MSCs, hUC-MSCs pre-treated with IL-6 for 48 h. Bars indicate the means and standard error of the mean from at least 3 independent experiments. Magnification, ×100; scale bar, 100 *μ*m; ^*^P<0.05.

The above data indicated that pre-treatment with IL-6 eliminated the promoting effect of hUC-MSCs on the migration and proliferation of gastric cancer cells *in vitro*. To confirm the role of IL-6-hUC-MSCs in gastric cancer cells *in vivo*, we co-injected SGC-7901 gastric cancer cells with hUC-MSCs or IL-6-hUC-MSCs into BALB/c nude mice to establish a subcutaneous tumor xenograft model of gastric cancer. SGC-7901 cells alone were transplanted as a control. Four weeks later, the xenograft tumors were removed, photographed and weighed. As shown in Fig. 5A, the size and weight of the tumors from the group co-injected with hUC-MSCs were evidently greater compared to the other 2 groups. The size and weight of the tumors in the group co-injected with IL-6-hUC-MSCs were similar to those in the control group (Fig. 5A-a and -b). H&E staining of the tumor tissue in each group revealed that the tissue structure was orderly and tightly organized in the control and hucMSC groups, whereas it was obviously loose and messy in the IL-6-hUC-MSC group (Fig. 5B).

Based on the above phenomenon, we wished to determine whether pre-treatment with IL-6 abolishes the growth-promoting effect of HUC-MSCs in gastric cancer through the inhibition of cell proliferation and the induction of cell apoptosis. We performed PCNA and TUNEL-based immunohistochemical staining on tissue sections from each group. The results revealed that the percentage of PCNA-positive cells in the IL-6-hUC-MSC group was markedly lower than that in the hucMSC group, wherease it was approximately equal to that in the control group (Fig. 5C). Apoptosis assay revealed that the percentage of apoptotic cells in the hUC-MSC group was the lowest (10%) among the 3 groups. However, the percentage of apoptotic cells in the IL-6-hUC-MSC group was reached 75% which was much higher than that in the control group (Fig. 5D). These data indicate that pre-treatment with IL-6 abolishes the growth-promoting effect of hUC-MSCs on gastric cancer cells.

## Discussion

Inflammatory conditions affecting the stomach are associated with an increased risk of cancer and progression. The hallmarks of cancer-associated inflammation mainly include the infiltration of cells of the microenvironment, cytokines, chemokines, growth factors and matrix-degrading enzymes (4). MSCs, which are considered to be the origin of tumor-associated fibroblasts, are emerging as one of the major components of the tumor microenvironment (23,24). Previous studies have demonstrated that MSCs are capable of migrating to primary tumor sites, where the tumor-associated inflammatory environment converts newly arrived MSCs into tumor-resident MSCs that display distinct properties, particularly a strong tumor-promoting activity (25,26). Our group firstly isolated GC-MSCs and GCN-MSCs from gastric tissues with different inflammatory conditions and found that GC-MSCs exhibited a greater capacity to promote the growth of gastric cancer (9). The abovementioned data suggest that cancer-related inflammation is an important factor contributing to the of MSCs to be ‘educated’ as tumor stromal cells.

IL-6, as an inflammatory cytokine, does not only participate in the communication between cells, but is also involved in carcinogenesis (27). Based on the function of IL-6 in inflammation-related carcinogenesis, we focused on the role of IL-6 in the conversion of hUC-MSCs into tumor-supporting cells.

CAFs, as classic stromal cells in the tumor microenvironment, have been extensively investigated and may originate from MSCs (29). The overexpression of α-SMA and cancer-promoting inflammatory cytokines is normally used to define the CAF-like phenotype (6). In order to analyze the phenotype of hUC-MSCs pre-treated with IL-6, we focused on α-SMA and inflammatory cytokines. Our results revealed that α-SMA expression was significantly induced in the hUC-MSCs by pretreatment with IL-6. STAT3, as a downstream effector of IL-6, was phosphorylated (its expression was analyzed to confirm the role of treatment with IL-6). The phenotype of CAFs suggests that hUC-MSCs may be activated by IL-6 superficially. However, when we examined the mRNA levels and supernatant content of inflammatory cytokines in hUC-MSCs and IL-6-hUC-MSCs, we found that the levels of several cancer-promoting cytokines, including CCL5, PDGF-BB, MCP-1 and TNFα, were markedly downregulated in the IL-6-hUC-MSCs. CCL5 is involved in the cross-talk between breast cancer cells and MSCs. Breast cancer cells stimulate CCL5 secretion by MSCs, and CCL5 in turn mediates MSC-induced cancer cell migration and invasion (30,31). Furthermore, ovarian cancer cells have been shown to reprogram normal fibroblasts into becoming CAFs through the downregulation of miR-214, which increased the production of CCL5 and endowed fibroblasts with tumor-promoting properties (32). Ren *et al* (25) compared the cytokine profiles between MSCs isolated from spontaneous lymphomas (L-MSCs) and bone marrow-derived MSCs (BM-MSCs) and found that MCP-1 expression was significantly increased in the supernatant of L-MSCs. MCP-1 is important for the recruitment of macrophages or monocytes by tumor-educated MSCs in promoting tumor development (25,34). TNFα is the prototypical pro-inflammatory cytokine. Inflammatory cells in the tumor microenvironment can produce TNFα. TNFα signaling can promote cell survival, invasion and angiogenesis (33). PDGF-BB has been demonstrated to modulate endothelial cell proliferation and tumor angiogenesis (34). The downregulation of the above cytokines indicates that pre-treatment with IL-6 impairs the cross-talk of hUC-MSCs with tumor cells or other cells of the tumor microenvironment.

In this study, the secretion of cytokines by IL-6-hUC-MSCs was suppressed, whereas only the secretion of IL-6 was induced. We confirmed that the increase in IL-6 secretion was not due to the contamination of recombinant IL-6 protein. We inferred that pre-treatment with IL-6 may induce hUC-MSCs to secrete IL-6. We also wished to determine whether hUC-MSCs pretreated with IL-6 promote the development of gastric cancer. In order to reveal the role of IL-6-pre-treated hUC-MSCs in gastric cancer, we co-cultured MNNG-transformed precancerous GES-1 cells or SGC-7901 gastric cancer cells with hUC-MSCs or IL-6-hUC-MSCs. We found that the hUC-MSCs evidently promoted the proliferation and migration of GES-1 and SGC-7901 cells. However, following treatment with IL-6, the hUC-MSCs did not have a growth-promoting effect on gastric epithelial and cancer cells. To further evaluate the altered function of hUC-MSCs pre-treated with IL-6 *in vivo*, we co-transplanted the MSCs with SGC-7901 gastric cancer cells into nude mice. Consistent with our *in vitro* results, the hUC-MSCs significantly promoted gastric cancer growth. There were no obvious differences between the control and the IL-6-hUC-MSC group. Pathological and histochemical analysis indicated that the hUC-MSCs promoted gastric cancer growth by enhancing the proliferation ability of the cancer cells and inhibiting apoptosis. On the contrary, pre-treatment with IL-6 endowed the hUC-MSCs with the functional properties of the inhibition of cell proliferation and the induction of cell apoptosis.

These data indicate that hUC-MSCs pre-treated with IL-6 do not promote gastric cancer progression and that their growth-promoting effect on gastric cancer is abolished. Analysis of the expression profiles of cytokines revealed that, regardless of the IL-6 levels being increased in the IL-6-hUC-MSCs, this did not induce a growth-promoting effect on gastric cancer. We inferred that hUC-MSCs promote gastric cancer progression through the synergistic action of inflammatory cytokines. It has been suggested that the NF-κB pathway plays a key role in tumor-infiltrating inflammatory cells (1). NF-κB activation in these cells leads to the secretion of pro-inflammatory cytokines. In the present study, NF-κB, as a key inflammatory transcriptional factor, was found to be inactivated in hUC-MSCs stimulated with IL-6; this provides a possible explanation for the fact that several cancer-promoting cytokines were suppressed in the supernatant from hUC-MSCs pre-treated with IL-6. Moreover, in a previous study of ours, we demonstrated that NF-κB activation was necessary for *H. pylori* to induce the differentiation of hUC-MSCs into CAFs (13). A previous study demonstrated that the inhibition of NF-κB activation in the tumor microenvironment represents a potentially effective strategy for arresting tumor growth (35). This finding suggests that NF-κB inactivation blocks the conversion of hUC-MSCs by IL-6 into tumor-supporting cells.

Although we found that stimulation with IL-6 altered the growth-promoting effect of hUC-MSCs on gastric cancer cells *in vitro* and *in vivo*, the mechanism through which this process occurs remains unclear. Future research is required to clarify the mechanism and study the role of IL-6-pretreated hUC-MSCs in other types of cancer.

The present study focused on the phenotype of IL-6-pretreated hUC-MSCs and their effects on gastric epithelial cells. We found that pre-treatment with IL-6 significantly abolished the promoting effect of hUC-MSCs on the proliferation and migration of gastric epithelial cells. The findings of this study provide new insight into the role of the inflammatory cytokine, IL-6, in the tumor-promoting effects of MSCs and its function in gastric cancer.

## Figures and Tables

**Table I:** Sequences of primers used for RT-qPCR and the conditions for amplification.

Genes	Primers sequences (5′-3′)	Annealing temperature (˚C)	Product length (bp)
MCP-1	F: GAACCGAGAGGCTGAGACTA		
	R: GCCTCTGCACTGAGATCTTC	59	259
CCL5	F: GGATTCCTGCAGAGGATCAA		
	R: GTGGTGTCCGAGGAATATGG	62	154
IL-6	F: TACATCCTCGACGGCATCTC		
	R: AGCTCTGGCTTGTTCCTCAC	61	252
IL-8	F: GCTCTGTGTGAAGGTGCAGTTT		
	R: TTCTGTGTTGGCGCAGTGT	62	144
β-actin	F: CACGAAACTACTCCCAACTCC		
	R: CATACTCC TGCTTGAGCTGATC	56	265

F, forward primer; R, reverse primer. MCP-1, monocyte chemoattractant protein-1; CCL5, chemokine (C-C motif) ligand 5; IL, interleukin.
